# Clinician Knowledge and Attitudes About Climate Change and Health After a Quality Incentive Program

**DOI:** 10.1001/jamanetworkopen.2024.26790

**Published:** 2024-08-08

**Authors:** Wynne Armand, Michael Padget, Elizabeth Pinsky, Jason H. Wasfy, Jonathan E. Slutzman, Ann-Christine Duhaime

**Affiliations:** 1 Center for the Environment and Health, Massachusetts General Hospital, Boston; 2 Division of General Internal Medicine, Department of Medicine, Harvard Medical School, Boston, Massachusetts; 3 Department of Psychiatry, Harvard Medical School, Boston, Massachusetts; 4 Division of Cardiology, Department of Medicine, Harvard Medical School, Boston, Massachusetts; 5 Mongan Institute, Massachusetts General Hospital, Boston; 6 Massachusetts General Physicians Organization, Boston; 7 Department of Emergency Medicine, Massachusetts General Hospital and Harvard Medical School, Boston; 8 Department of Pediatric Neurosurgery, Harvard Medical School, Boston, Massachusetts

## Abstract

**Importance:**

Climate change is a fundamental threat to human health, and industries, including health care, must assess their respective contribution to this crisis.

**Objective:**

To assess the change in knowledge of clinicians who completed a quality incentive program (QIP) measure on climate change and health care sustainability and to examine clinician attitudes toward climate change and their perception of clinical and individual relevance.

**Design, Setting, and Participants:**

The participants in this survey study included employed physicians and psychologists who were part of a hospital physician organization in an academic medical center (AMC) in Boston, Massachusetts. The hospital physician organization provides a QIP with different measures every 6 months and provides incentive payments on completion. The study is based on a survey of participants on completion of a QIP measure focused on climate change and health care sustainability offered from July 2023 through September 2023 at the AMC.

**Exposure:**

Structured educational video modules.

**Main Outcomes and Measures:**

After completion of the modules, the participants reported their baseline and postintervention knowledge on climate change impacts on health and health care sustainability, perceived relevance of the material, and attitudes toward the modules using 5-point Likert scales and free-text comments. Data were analyzed using univariate and multivariable analyses including participant age, gender, and practice specialty.

**Results:**

Of the 2559 eligible clinicians, 2417 (94.5%) (mean [SD] age, 48.9 [11.5] years; range, 29-85 years; 1244 males [51.5%]) participated in the measure and completed the survey. Among these participants, 1767 (73.1%) thought the modules were relevant or very relevant to their lives and 1580 (65.4%) found the modules relevant or very relevant to their clinical practice. Age was not associated with responses. Practitioners in specialties classified as climate facing were more likely to think that the education was relevant to their clinical practice compared with those in non–climate-facing specialties (mean [SD] score, 3.76 [1.19] vs 3.61 [1.26]; *P* = .005). Practitioners identifying as female were also more likely to consider this education as relevant to their clinical practice compared with male practitioners (mean [SD] score, 3.82 [1.17] vs 3.56 [1.27]; *P* < .001).

**Conclusions and Relevance:**

In this survey study, a high proportion of clinicians expressed positive attitudes toward education in climate change and health and health care sustainability, with some demographic and specialty variability. These data support that climate and health education in AMCs provides information that practitioners see as relevant and important.

## Key Points

**Question:**

Can a quality incentive program (QIP) measure for clinicians change their perceived knowledge about climate change’s health impacts and health care sustainability?

**Findings:**

In this survey study of 2417 clinicians who completed a QIP measure about climate change and health care sustainability at an academic medical center, most felt these topics are relevant to their practices and reported an increase in their knowledge after completion of the measure.

**Meaning:**

The findings of this study indicate that tailored strategies may engage clinicians in learning about climate change’s health effects and mitigation of health care sector contributions.

## Teaser

This survey study examines clinicians’ knowledge of and attitudes toward climate change and their perception of clinical relevance before and after a quality incentive program about the impact of climate change on health and health care sustainability.

## Introduction

Climate change is a fundamental threat to human health and is recognized by many as the greatest health crisis humans have ever faced.^1^ The most recent United Nations Framework Convention on Climate Change Conference of the Parties (COP28) held the first-ever Health Day to address climate change’s health impacts, the climate resiliency of health systems, and adaptation measures to decrease the impact on human health.^2^ As all industries examine their respective contributions to this crisis, the White House/HHS Health Sector Climate Pledge asks organizations to join the commitment to reducing emissions and improving climate resilience.^3^ As institutions strategize on how to achieve these targets, education of physicians is a foundational lever for reaching these goals, given their roles throughout the strata of patient care and trainee education at academic medical centers (AMCs).

While surveys demonstrate that most US residents are alarmed or concerned about global warming, with differences in attitudes based on age, gender, and race, little is known about attitudes among physicians.^4^ In a 2023 convenience sample of 1001 clinicians, 79% felt it was important that their organization address climate change and minimize its environmental impact; 75% felt it was important that as individuals they do likewise at work.^5^ However, this study did not systematically survey medical staff and was not able to describe differences in opinion within medical specialties.

Despite the importance, surveys show that the majority of physicians do not feel prepared to address climate change’s impact on health or to take action.^6,7^ There are limited data on the most effective way to train clinicians on these issues, which may vary depending on individual knowledge base, specialty, or attitudes.

Here, we describe educational modules offered at an academic medical center in Boston (Massachusetts General Hospital) through an existing biannual physician quality incentive program (QIP). The modules served to provide a foundation about climate change and fossil fuel–related pollution’s impact on health, the health care system’s contribution to carbon pollution, and opportunities for mitigation. To our knowledge, this is the first undertaking to provide a hospital-wide climate and sustainability educational opportunity for physicians through a QIP at an AMC.

Our primary goal was to assess how educational modules might affect knowledge on these topics among physicians. The secondary goals were to explore physicians’ attitudes toward climate change and health care sustainability, and their associations with demographic and professional variables. Specifically, we assessed if age, self-identified gender, or specialty influenced attitudes about perceived individual and clinical relevance of climate change and sustainability education or tone of feedback comments. Our expectation was that these results could further direct how to best influence clinician perspectives and behavior in future educational and outreach efforts.

## Methods

### Survey Study

This survey study was conducted at a single time point at module completion, and participants were asked at that point to estimate both their premodule baseline and postmodule knowledge levels. Data on race and ethnicity were not collected because this information is not typically required for biannual physician organization (PO) QIP measures. In addition, some departments are small and revealing these data could identify individual respondents. The modules and assessments were a part of a QIP and did not require approval as decided by the Massachusetts General Brigham Institutional Review Board. Informed consent was not obtained as the survey results were not reviewed with participant identifiers. The study followed the American Association for Public Opinion Research (AAPOR) reporting guideline.

### Setting

Our hospital is an approximately 1000-bed AMC in Boston, Massachusetts, encompassing inpatient, outpatient, research, and administrative functions. The hospital supports a Center for the Environment and Health (hereafter, Center), which is responsible for increasing environmental considerations in all aspects of AMC operations, including education. The Center also initiated the current project.^8^

Since 2006, the hospital PO has offered a QIP for its salaried physicians and psychologists.^9^ Every 6 months, 3 new measures are offered to eligible clinicians with incentive payments on completion. Examples of past QIP measures include electronic health record training, hand hygiene compliance, and physician communication training. The QIP is funded by an existing administrative fee that is a percentage of practice expenses covering PO services and management functions. During July to September 2023, 1 of the 3 offered QIP measures was a collection of learning modules to provide a foundation of knowledge on the effects of climate change on health as well as health care sustainability.

### QIP Design

Clinical leaders at the Center created a series of narrated slide decks. These recorded presentations were hosted on YouTube and then integrated into learning modules accessible through the Qualtrics Experience Management platform (Qualtrics). Participant selection of their clinical department or division (eTable 1 in Supplement 1) directed which track they would receive of the recorded presentations on the Qualtrics platform. All participant tracks included the following general information: (1) climate change impacts on health (reviewing how different climate exposure pathways and pollution affect health); (2) health care sector’s environmental footprint (scope 1-3 greenhouse gas emissions, breakdown of US health care carbon footprint, examples of waste audits, and life cycle assessments); (3) current hospital-wide pro-environmental actions; and (4) suggestions for taking action (including personal choices, data on academic travel, and opportunities for advocacy). Interspersed with this general information were 4 assessment questions on content to keep participants engaged. Along with the 4 standard tracks, additional customized presentations were included for the following departments: Anesthesia, Emergency Medicine/Urgent Care, Pathology, Psychiatry, Pediatrics, Primary Care, Obstetrics and Gynecology (OB GYN), Radiology, Radiation Oncology, and Surgery and surgical subspecialties. For example, anesthesiologists received information on anesthetic greenhouse gases; pediatricians, on how children experience climate change and pollution differently than adults; and radiologists, on the energy use of imaging equipment. Although the duration of the presentations varied somewhat by specialty, most were approximately 30 minutes long for all components.

The PO QIP home page (eFigure 2 in Supplement 1) introduced the role physicians have in transforming health care delivery to mitigate its carbon footprint and ensure the well-being of patients. Links to additional resources and specialty modules were provided.

### Participants

The QIP is offered to clinically active nontrainee physicians and psychologists who participate in managed care contracts with major local insurance companies. Participants have 3 months to complete their 3 measures. The amount of payment to participants on completion of each measure depends on the level of their clinical activity, divided into 3 tiers: $833.33, $416.67, or $166.67 per completed measure.

### Survey Data Collection

After finishing the educational modules, participants completed the required survey questions (eFigure 1 in Supplement 1) from within the Qualtrics platform. Only then was participation confirmed for credit. Participants were asked to rank, using a 5-point Likert scale, how relevant the material was to them as individuals and in their clinical practice (1 indicates very irrelevant; 2, somewhat irrelevant; 3, neutral; 4, somewhat relevant; and 5, very relevant). They were also asked to rank their knowledge level on climate change’s impacts on health and health care sustainability before and after completing the modules (1 indicates very low; 2, low; 3, neutral; 4, high; and 5, very high). Participants were also able to provide free-text comments.

Participant data available included age, gender, and specialty (department/division). Data were analyzed without participant identifiers.

### Specialty and Comment Categorization

Based on the investigators’ observations in interacting with clinicians across specialties on issues of climate change and health, we hypothesized that those in predominantly procedure-based specialties were less likely to perceive climate change as relevant to their practices. Conversely, some specialties and professional organizations have been more invested in climate change and health because of immediate relevance to conditions treated in those specialties. To explore the association of specialty with participant responses, each specialty was coded (Table 1) by consensus of the investigators as being predominantly procedural (yes or no) or as having professional contact with the direct health impacts of climate change, that is, being a “climate-facing” specialty (yes or no). Specialties were categorized along these 2 dimensions independently.

**Table 1.** Respondent and Department Characteristics and Reported Outcomes

Department	Clinicians, No.	Age, mean (SD), y	Gender, No. (%)		Procedural specialty	Climate facing^a^	Score, mean (SD)				
			Male	Female			Relevance: individual (1-5)	Relevance: clinical (1-5)	Subject knowledge before (1-5)	Subject knowledge after (1-5)	Knowledge change
Anesthesia	163	47.8 (11.1)	103 (63.2)	60 (36.8)	Yes	No	3.88 (1.39)	3.85 (1.39)	3.34 (0.80)	3.91 (0.83)	0.57
Dermatology	43	48.9 (13.1)	21 (48.8)	22 (51.2)	No	Yes	3.65 (1.29)	3.40 (1.20)	2.91 (0.72)	3.74 (0.62)	0.83
Emergency	62	46.4 (9.6)	39 (62.9)	23 (37.1)	No	Yes	4.05 (1.30)	3.82 (1.30)	3.23 (0.97)	3.85 (0.88)	0.62
Medicine–Allergy	14	49.2 (11.7)	5 (35.7)	9 (64.3)	No	Yes	4.14 (0.95)	3.86 (1.17)	3.64 (0.50)	4.00 (0.88)	0.36
Medicine–Belmont IMA	8	58.0 (8.8)	7 (87.5)	1 (12.5)	No	Yes	4.13 (0.83)	3.88 (1.13)	3.25 (1.16)	3.75 (0.71)	0.50
Medicine–Cardiology	103	50.6 (11.7)	71 (68.9)	32 (31.1)	No	No	3.99 (1.29)	3.75 (1.15)	3.10 (0.89)	4.00 (0.73)	0.90
Medicine–Core Educators	7	48.9 (10.4)	5 (71.4)	2 (28.6)	No	Yes	3.86 (1.46)	3.86 (1.46)	2.71 (0.76)	3.57 (0.53)	0.86
Medicine–Endocrine	60	50.1 (12.0)	24 (40)	36 (60)	No	No	3.87 (1.36)	3.62 (1.21)	2.98 (1.05)	3.93 (0.73)	0.95
Medicine–Gastrointestinal	51	49.0 (11.9)	32 (62.8)	19 (37.2)	No	No	3.88 (1.16)	3.73 (1.27)	3.25 (0.98)	3.88 (0.84)	0.63
Medicine–Hematology Oncology	128	48.5 (10.6)	79 (61.7)	49 (38.3)	No	No	3.84 (1.32)	3.45 (1.25)	3.23 (0.89)	3.93 (0.69)	0.70
Medicine–Hospital Medicine Unit	89	38.3 (7.8)	42 (47.2)	47 (52.8)	No	Yes	4.24 (1.00)	3.98 (1.04)	3.04 (0.94)	3.83 (0.71)	0.79
Medicine–Infectious Disease	57	49.9 (9.7)	27 (47.4)	30 (52.6)	No	Yes	4.11 (1.21)	3.68 (1.10)	3.28 (0.80)	3.77 (0.87)	0.49
Medicine–Nephrology	29	49.4 (14.3)	17 (58.6)	12 (41.4)	No	No	3.69 (1.42)	3.45 (1.35)	3.07 (0.92)	4.00 (0.71)	0.93
Medicine–Palliative Care	27	46.2 (9.4)	7 (25.9)	20 (74.1)	No	No	3.81 (1.36)	3.37 (1.39)	2.96 (0.81)	3.89 (0.51)	0.93
Medicine–Primary Care	203	51.7 (11.4)	74 (36.4)	129 (63.6)	No	Yes	4.15 (1.19)	3.91 (1.13)	3.27 (0.88)	4.02 (0.76)	0.75
Medicine–Pulmonary & Critical Care	59	48.1 (12.4)	33 (55.9)	26 (44.1)	No	Yes	3.95 (1.32)	3.64 (1.27)	3.29 (0.87)	3.73 (0.85)	0.44
Medicine–Rheumatology	19	48.0 (12.2)	10 (52.6)	9 (47.4)	No	No	4.47 (0.61)	3.95 (0.78)	2.53 (0.96)	3.74 (0.73)	1.21
Medicine–Urgent Care	12	49.7 (12.4)	4 (33.3)	8 (66.7)	No	Yes	4.42 (1.00)	4.00 (1.21)	3.00 (0.95)	4.08 (0.67)	1.08
Neurology	128	48.9 (10.9)	72 (56.2)	56 (43.8)	No	No	3.67 (1.40)	3.48 (1.18)	3.16 (0.91)	3.80 (0.75)	0.64
Neurosurgery	23	53.0 (12.5)	20 (87.0)	3 (13.0)	Yes	No	3.96 (1.11)	3.83 (0.98)	3.61 (0.78)	3.78 (0.95)	0.17
Obstetrics and Gynecology	69	49.0 (10.6)	12 (17.4)	57 (82.6)	Yes	Yes	4.20 (1.37)	4.12 (1.39)	3.35 (1.04)	4.26 (0.63)	0.91
Orthopedics	55	49.4 (10.7)	49 (89.1)	6 (10.9)	Yes	No	3.44 (1.34)	3.27 (1.33)	2.80 (1.01)	3.58 (0.96)	0.78
Pathology	64	52.4 (11.7)	32 (50.0)	32 (50.0)	No	No	3.72 (1.35)	3.42 (1.31)	3.14 (0.81)	3.97 (0.69)	0.83
Pediatric Surgical Services	7	58.1 (13.6)	4 (57.1)	3 (42.9)	Yes	No	4.29 (0.95)	4.14 (0.90)	3.43 (0.53)	3.71 (0.76)	0.28
Pediatrics	219	51.2 (11.8)	73 (33.3)	146 (66.7)	No	Yes	3.98 (1.28)	3.76 (1.13)	3.05 (0.92)	3.96 (0.78)	0.91
Physical Medicine & Rehabilitation	62	46.8 (10.8)	34 (54.8)	28 (45.2)	No	No	3.65 (1.33)	3.50 (1.22)	3.02 (1.00)	3.79 (0.83)	0.77
Psychiatry	351	47.5 (11.8)	138 (39.3)	213 (60.7)	No	Yes	4.00 (1.28)	3.61 (1.20)	2.96 (0.96)	3.96 (0.78)	1.00
Radiation Oncology	32	48.1 (9.2)	17 (53.1)	15 (46.9)	No	No	4.16 (0.95)	3.81 (0.97)	3.16 (0.99)	4.03 (0.74)	0.87
Radiology	131	48.1 (11.4)	91 (69.5)	40 (30.5)	No	No	3.72 (1.40)	3.60 (1.33)	3.27 (1.00)	3.97 (0.82)	0.70
Surgery	126	50.8 (10.8)	89 (70.6)	37 (29.4)	Yes	No	3.81 (1.32)	3.66 (1.27)	3.33 (1.03)	3.87 (0.92)	0.54
Urology	16	54.4 (13.1)	13 (81.3)	3 (18.7)	Yes	No	4.13 (1.50)	3.81 (1.42)	3.13 (1.09)	3.94 (1.06)	0.81
Total	2417	48.9 (11.5)	1244 (51.5)	1173 (48.5)	NA	NA	3.93 (1.29)	3.69 (1.23)	3.14 (0.93)	3.92 (0.79)	0.78

Abbreviations: IMA, Internal Medicine Associates; NA, not applicable.

^a^ Indicates if the specialty has direct contact with health effects of climate change.

Comments provided in the free-text field were classified by comment tone into positive, negative, or neutral categories and confirmed by 2 investigators. Comment tone was identified independent of the subject of the comment. Comments suggesting targets for intervention without specific positive comments were classified as neutral even if these comments suggested an active engagement with the material. Positive comments included statements such as, “This education was very helpful in learning about this important topic and I’d like to learn more.” Negative comments described the modules as not being relevant or important. Neutral comments included suggestions for institutional changes, health care sustainability suggestions, or other comments that did not specifically address the utility or value of the educational content.

### Statistical Analysis

Univariate and multivariable logistic regression models were used to explore the associations between questionnaire responses (individual relevance, clinical relevance, comment tone) with age, gender, and specialty. All models also were repeated without including responses from anesthesiologists, as this specialty did not fit into either dichotomous procedural or climate-facing categories and because anesthesiologists in our hospital had recently received extensive education on the greenhouse gas effects of volatile anesthetics. Models testing associations with comment tone were limited to participants who included a comment. Interactions were tested in each model by including interaction terms for gender and climate-facing specialty and gender and procedural specialty variables. Comment tone was coded as negative (1), neutral (2), or positive (3). Analyses were completed using SAS System for Windows software, version 9.4 (SAS Institute Inc). The threshold for statistical significance set at *P* = .05 (2-tailed). These analyses were exploratory, and no adjustments were made to control for multiple comparisons.

## Results

Of the total 2559 eligible physicians (95%) and psychologists (5%), 2417 (94.5%) completed these educational modules, a similar completion rate compared with past QIP measures. Of these, the mean (SD) age was 48.9 (11.5) years (range, 29-85 years); 1244 (51.5%) were males and 1173 (48.5%) were females. Since all survey questions had to be answered to be considered complete (with free-text comment optional), there were no missing participant data.

The number of participants varied substantially by department or division, ranging from 7 in both Medicine–Core Educators and Pediatric Surgical Services, to 351 in Psychiatry. The proportion of male participants varied by department, from 12 (17.4%) in OB GYN to 49 (89.1%) in Orthopedics. Seven of the 31 specialties were classified as being procedural, which included 459 respondents (19.0%). Thirteen of the 31 specialties were classified as climate-facing, which included 1193 respondents (49.4%) (Table 1).

Among the total participants, 1767 (73.1%) thought the modules were relevant or very relevant to their lives and 1580 (65.4%) found the modules relevant or very relevant to their clinical practice. Age was not associated with responses. Practitioners in specialties classified as climate facing were more likely to think that the education was relevant to their clinical practice compared with those in non–climate-facing specialties (mean [SD] score, 3.76 [1.19] vs 3.61 [1.26]; *P* = .005). Practitioners identifying as female were also more likely to consider this education as relevant to their clinical practice compared with male practitioners (mean [SD] score, 3.82 [1.17] vs 3.56 [1.27]; *P* < .001).

The mean (SD) individual relevance score (ie, degree to which the material was judged as relevant to the clinician’s individual life) was 3.93 (1.29) on the 5-point Likert scale. The division or department with the lowest mean (SD) individual relevance score was Orthopedics (3.44 [1.34]) and that with the highest score was Medicine–Rheumatology (4.47 [0.61]).

The mean (SD) clinical relevance score (ie, relevance to the clinician’s professional practice) was 3.69 (1.23) (Table 1). The division or department with the lowest clinical relevance score was Orthopedics (3.27 [1.33]), while that with the highest was Pediatric Surgical Services (4.14 [0.90]).

The mean (SD) self-reported subject knowledge score before the training was 3.14 (0.93), with Orthopedics having the lowest score (2.80 [1.01]) and Pediatric Surgical Services, the highest (3.43 [0.53]). The mean (SD) self-reported subject knowledge score after the training was 3.92 (0.79), with Medicine–Core Educators having the lowest score (3.57 [0.53]) and OB GYN, the highest (4.26 [0.63). The mean change in knowledge score was 0.78, with Neurosurgery having the lowest score (0.17) and Medicine–Rheumatology having the highest (1.21).

Of all participants, 446 (18.5%) provided optional comments, of which 250 (56.1%) were classified as positive; 163 (36.5%), neutral; and 33 (7.4%), negative (Table 2). Many positive comments highlighted the importance of the topic and included suggestions for improving the carbon footprint of the hospital. Negative comments included those from respondents who felt that quality improvement trainings should be directed toward patient care or that providing climate information to clinicians was inappropriate given their limited influence on hospital-wide policy.

**Table 2.** Participant Characteristics by Comment Tone (Univariate Analysis)

Characteristic	Negative (n = 33)	Neutral (n = 163)	Positive (n = 250)	*P* value
Age, mean (SD), y	51.8 (12.0)	52.4 (12.2)	52.6 (12.5)	.72
Male gender, No. (%)	23 (69.7)	62 (38.0)	131 (52.4)	.66
Female gender, No. (%)	10 (30.3)	101 (62.0)	119 (47.6)	.66
Nonprocedural, No. (%)	25 (75.8)	130 (79.8)	214 (85.6)	.06
Climate-facing, No. (%)	12 (36.4)	95 (58.3)	125 (50.0)	.98

### Univariate Analyses

In univariate analyses (Table 2 and Table 3), female gender and practicing in a climate-facing specialty were associated with greater individual relevance and also were significantly associated with higher reported relevance to participants’ clinical practice. None of the tested variables were associated significantly with comment tone in univariate analyses.

**Table 3.** Participant Characteristics by Level of Reported Relevance (Univariate Analysis)

Characteristic	Very irrelevant	Somewhat irrelevant	Neutral	Somewhat relevant	Very relevant	*P* value
**Individual relevance**						
No. of respondents	229	157	264	669	1098	NA
Age, mean (SD), y	50.5 (11.3)	48.2 (12.1)	48.3 (10.5)	47.6 (11.3)	49.6 (11.8)	.86
Male gender, No. (%)	138 (60.3)	94 (59.9)	156 (59.1)	350 (52.3)	506 (46.1)	<.001
Female gender, No. (%)	91 (39.7)	63 (40.1)	108 (40.9)	319 (47.7)	592 (53.9)	<.001
Nonprocedural, No. (%)	178 (77.7)	127 (80.9)	206 (78.0)	562 (84.0)	885 (80.6)	.29
Climate facing, No. (%)	96 (41.9)	72 (45.9)	101 (38.3)	332 (49.6)	592 (53.9)	<.001
**Clinical relevance**						
No. of respondents	196	252	389	855	725	NA
Age, mean (SD), y	50.8 (11.6)	48.4 (11.7)	48.6 (11.2)	48.6 (11.6)	49.1 (11.6)	.37
Male gender, No. (%)	123 (62.8)	147 (58.3)	210 (54.0)	436 (51.0)	328 (45.2)	<.001
Female gender, No. (%)	73 (37.2)	105 (41.7)	179 (46.0)	419 (49.0)	397 (54.8)	<.001
Nonprocedural, No. (%)	146 (74.5)	217 (86.1)	317 (81.5)	740 (86.5)	538 (74.2)	.10
Climate facing, No. (%)	81 (41.3)	120 (47.6)	168 (43.2)	455 (53.2)	369 (50.9)	.003

Abbreviation: NA, not applicable.

### Multivariable Analyses

Six multivariable models were run to test independent associations between participant and specialty characteristics and outcomes. (Full model results are given in eTable 2 in Supplement 1.) The tested interaction effects were nonsignificant and not included in the final models.

A multivariable model was constructed to test associations with reported individual relevance. In the model, which included gender, age, climate-facing, and procedural specialty statuses as independent variables, both female gender (mean score increase vs male 0.25; 95% CI, 0.14-0.35) and practicing in a climate-facing specialty (mean score increase vs non–climate-facing specialty, 0.19; 95% CI, 0.08-0.30) showed significant associations with higher reported individual relevance. No differences in associations were found when these models were repeated without anesthesiologists’ responses.

A multivariable model including gender, age, climate-facing specialty, and procedural specialty statuses as independent variables was also run to test associations with reported relevance to participants’ clinical practice. Female gender (mean score increase vs male, 0.24; 95% CI, 0.14-0.35), climate-facing specialty (mean score increase vs non–climate-facing specialty, 0.15; 95% CI, 0.04-0.26), and procedural specialty (mean score increase vs nonprocedural specialty, 0.20; 95% CI, 0.07-0.33) were significantly associated with higher reported clinical relevance. This model repeated without anesthesiologists’ responses showed similar associations, with the exception of procedural specialty, which was no longer significantly associated with higher reported clinical relevance.

Last, a similar model was run to test associations with comment tone. In this model, working in a procedural specialty (mean score decrease vs nonprocedural specialty, 0.16; 95% CI, 0.002-0.32) was associated with more negative comment tones. No changes in associations were found when these models were repeated without anesthesiologists’ responses. In all of these models, age was not significantly associated with any of the outcomes.

## Discussion

While there is some early experience with integrating climate curricula into undergraduate and graduate medical education, there is little experience with integrating such training broadly into physician faculty development.^10,11,12,13^

This survey study’s educational modules on climate change’s health impacts and health care sustainability delivered to physicians and psychologists through a QIP measure was a novel intervention that was well received with a high completion rate, after which participants reported an increase in subject-matter knowledge. A majority also expressed appreciation for the educational modules and for institutional efforts on these issues in optional free-text comments. In addition, many clinicians who had positive responses to the training suggested in their comments additional ways to integrate more learning into their clinical practice environment. The minority of clinicians with predominantly negative comments did not believe such information was relevant to their practices and thought other topics were more worthy of their investment of time.

In this analysis, age did not influence the degree to which clinicians thought climate and health education was relevant to their individual lives nor to their practices, in contrast to some studies in the general population.^14,15^ Gender, however, did influence both measures, consistent with other studies.^16^

There are few data to assess attitudes on environment and health among specialties. As hypothesized, practitioners of disciplines that specifically treat problems directly exacerbated by climate change were more likely to think such training was relevant to their practice. Clinicians in procedural practices were significantly more likely to feel this training had clinical relevance compared with those in nonprocedural specialties, although this was no longer the case when data were analyzed without including anesthesiologists’ responses. This post hoc analysis was performed because of the department’s prior educational initiatives and its provision of individual anesthesiologist’s greenhouse gas emissions based on use of volatile and intravenous anesthetics.^17^ For these reasons, while by convention Anesthesia is considered a procedural specialty, it is unique with respect to its carbon footprint in the health system and unique within our hospital with respect to professional education. Of note, clinicians from the Pediatric Surgical Services also had received education on this topic during the study year, which may have influenced that division’s high scores on climate relevancy.

The high completion rate of 94.5% suggests robustness of the results, encompassing a broad sample of these AMC-employed physicians and psychologists. Given that only 7.4% of the written comments were negative, we interpret this as an indication that the majority of our clinicians are encouraged by learning this material. Many comments asked for additional instruction and ways to get involved.

### Implications

These data suggest that for some clinicians, additional detailed education on interventions for environmentally sustainable health care delivery would be welcome, and this provides an opportunity for institutional action. Conversely, since more negative attitudes tended to cluster in the procedural specialties, educational efforts might require different strategies. Strategies shown in other contexts to facilitate culture change include identifying allies within the community itself, finding interventions that directly benefit the clinicians and patients (eg, financial savings, improved efficiency, short-term health benefits), and creating a systemwide culture that promotes the importance of climate change and health through effective leadership.

This study demonstrates that this type of training is successful and could be expanded to other health care workers and other hospitals while being mindful of differences in audience (including department or division) when doing so. As educational resources on climate change and health care sustainability continue to expand and become more accessible, such training can be more easily tailored and made scalable for other health care institutions.

### Limitations

This study has several limitations. Not every specialty or subspecialty had an additional customized module relevant to their practices. We would expect additional knowledge change and perceived relevance if tailoring could be provided for more specialties. Also, there may be recall bias about baseline knowledge and knowledge change when administering the survey questions at a single time point.

Clinicians were financially incentivized to complete the training. While this encouraged participation, it may limit the generalizability of this approach where financial incentives may not be available or environmental issues are not a high enough institutional priority. Last, there was no longitudinal follow-up with the clinicians to assess durability of reported knowledge and no measurement of actual behavior change arising from the knowledge.

## Conclusions

According to this survey study, these PO-wide QIP modules on climate change health effects and health care sustainability achieved high participation and found that most clinicians believe this information is relevant to their practices and welcome opportunities for action. Demographic and specialty variability in attitudes toward environment and health education suggest more tailored strategies to engage clinicians in climate and health education and mitigating contributions from health care.

As professional journals call for urgent action to address climate change, there is a parallel need to educate health professionals so that they can recognize and address the health risks of climate change and take steps to minimize their contribution to the problem. This PO-wide educational intervention was feasible, well received, and a successful early step in addressing this urgent need.

## Notes

Supplement 1. **eTable 1.** Participant Roles and Presentation Modules **eTable 2.** Multivariable Analyses **eFigure 1.** Survey Questions **eFigure 2.** MGPO QIP Home Page

Supplement 2.
Data Sharing Statement
