# Supplementary material for: Clinician Knowledge and Attitudes About Climate Change and Health After a Quality Incentive Program
Source: JAMA Netw Open. 2024 Aug 8;7(8):e2426790. doi: 10.1001/jamanetworkopen.2024.26790 (PMC11310827; doi:10.1001/jamanetworkopen.2024.26790)
Supplement: Supplement 1. — eTable 1. Participant Roles and Presentation Modules eTable 2. Multivariable Analyses eFigure 1. Survey Questions eFigure 2. MGPO QIP Home Page [file jamanetwopen-e2426790-s001.pdf]

Supplementary Online Content

Armand W, Padget M, Pinsky E, Wasfy JH, Slutzman JE, Duhaime AC. Clinician knowledge and attitudes about climate change and health after a quality incentive program. *JAMA Netw Open*. 2024;7(8):e2426790.
doi:10.1001/jamanetworkopen.2024.26790

eTable 1. Participant Roles and Presentation Modules

eTable 2. Multivariable Analyses

eFigure 1. Survey Questions

eFigure 2. MGPO QIP Home Page

This supplementary material has been provided by the authors to give readers additional information about their work.

© 2024 Armand W et al. *JAMA Network Open.*

eTable 1. Participant roles and presentation modules

Participant by department/division	Learning modules received
Dermatology, Allergy, Cardiology, Endocrine, GI, Hematology/Oncology, Hospital Medicine, Infectious Disease, Nephrology, Palliative Care, Pulmonary/Critical Care, Rheumatology, Neurology, Physical Medicine & Rehab	Standard
Anesthesia	Standard plus<br>• Section on Anesthesia's carbon footprint and steps taken at MGH for mitigation
Emergency Medicine (EM), Urgent Care	Standard plus<br>• Section on climate change's impact on health from perspective EM/Urgent Care
OB GYN	Standard plus<br>• Section on heat and pollution impact on pregnant individuals
OB GYN, primary care	Standard plus<br>• Section including example of Life Cycle Analysis (LCA) of single-use vs reusable specula
Pathology	Standard plus<br>• Section including example of Life Cycle Assessments related to Pathology and laboratory tests
Pediatrics	Standard plus<br>• Section on climate change's impact on health of children
Psychiatry	Standard plus<br>• Section on climate change's impact on mental health
Radiation Oncology	Standard plus<br>• Section including example of Life Cycle Assessments on different radiation treatment cycles with clinical equipoise
Radiology	Standard plus<br>• Section on Radiology's carbon footprint and steps taken at MGB for mitigation
Surgery and surgical subspecialties (Neurosurgery, Orthopedics, Pediatric Surgical Services, Urology)	Standard plus<br>• Section on climate change's impact on health from perspective of surgical specialties<br>• Section on perioperative carbon footprint

© 2024 Armand W et al. *JAMA Network Open.*

eTable 2. Multivariable analyses

Individual relevance - all specialties				
Parameter	Estimate	Standard Error	t Value	Pr > |t|
Intercept	3.380	0.158	21.34	<.001
Age	0.002	0.002	1.11	0.26
Gender (m=1, f=2)	0.247	0.054	4.53	<.001
Climate (non-climate facing = 0, climate facing =1)	0.193	0.056	3.41	<.001
Procedural (procedural = 0, non-procedural = 1)	-0.044	0.071	-0.63	0.53
**Individual relevance - excluding Anesthesia**				
Parameter	Estimate	Standard Error	t Value	Pr > |t|
Intercept	3.383	0.169	19.92	<.001
Age	0.003	0.002	1.13	0.26
Gender (m=1, f=2)	0.223	0.056	3.99	<.001
Climate (non-climate facing = 0, climate facing =1)	0.200	0.056	3.54	<.001
Procedural (procedural = 0, non-procedural = 1)	-0.023	0.082	-0.28	0.78
**Clinical relevance - all specialties**				
Parameter	Estimate	Standard Error	t Value	Pr > |t|
Intercept	3.418	0.150	22.71	<.001
Age	-0.00003	0.002	-0.02	0.99
Gender (m=1, f=2)	0.243	0.052	4.69	<.001
Climate (non-climate facing = 0, climate facing =1)	0.149	0.054	2.79	0.005
Procedural (procedural = 0, non-procedural = 1)	-0.202	0.067	-3.01	0.003
**Clinical relevance - excluding Anesthesia**				
Parameter	Estimate	Standard Error	t Value	Pr > |t|
Intercept	3.382	0.161	21.05	<.001
Age	0.0001	0.002	0.07	0.95

© 2024 Armand W et al. *JAMA Network Open.*

Gender (m=1, f=2)	0.218	0.053	4.12	<.001
Climate (non-climate facing = 0, climate facing =1)	0.162	0.053	3.03	0.002
Procedural (procedural = 0, non-procedural = 1)	-0.145	0.077	-1.88	0.06
**Comment tone - all specialties**				
Parameter	Estimate	Standard Error	t Value	Pr > |t|
Intercept	2.369	0.187	12.65	<.001
Age	0.0008	0.002	0.34	0.73
Gender (m=1, f=2)	-0.035	0.063	-0.56	0.58
Climate (non-climate facing = 0, climate facing =1)	-0.018	0.064	-0.29	0.77
Procedural (procedural = 0, non-procedural = 1)	0.163	0.082	2	0.05
**Comment tone - excluding Anesthesia**				
Parameter	Estimate	Standard Error	t Value	Pr > |t|
Intercept	2.353	0.197	11.93	<.001
Age	0.001	0.002	0.22	0.83
Gender (m=1, f=2)	-0.043	0.064	-0.66	0.51
Climate (non-climate facing = 0, climate facing =1)	-0.010	0.064	-0.16	0.88
Procedural (procedural = 0, non-procedural = 1)	0.202	0.090	2.23	0.03

Comment tone code: negative=1, neutral=2, or positive=3

© 2024 Armand W et al. *JAMA Network Open.*

eFigure 1. Survey questions

How relevant do you feel this material is:

	Very irrelevant	Somewhat irrelevant	Neutral	Somewhat relevant	Very relevant
For you as an individual?	○	○	○	○	○
For your clinical practice?	○	○	○	○	○

Overall Knowledge Check

	Very Low	Low	Neutral	High	Very High
What was your overall knowledge level on climate impact on health and healthcare sustainability <u>before</u> taking this module?	○	○	○	○	○

	Very Low	Low	Neutral	High	Very High
How has your overall knowledge on climate impact on health and healthcare sustainability changed <u>after</u> taking this module?	○	○	○	○	○

Do you have comments or specific suggestions regarding this climate and healthcare sustainability QI module?

© 2024 Armand W et al. *JAMA Network Open.*

eFigure 2. MGPO QIP home page

Improving Your Results

The following documents provide best practices and FAQs to help you improve results on your measures for Term 2, 2023. For resources from previous measures, click here.

Mass General Hospital Center for the Environment and Health Resources
Climate change and other forms of pollution are greatly impacting our patients, especially those already most vulnerable. Meanwhile, the U.S. healthcare system contributes more to the climate crisis than any other nation's healthcare industry and is also a significant contributor to total domestic greenhouse gas emissions (8.5%). The healthcare system must work aggressively to reduce its environmental impact.

Physicians are integral to decision-making related to patient care, healthcare delivery, and biomedical research. Specifically, physicians are care team leaders, drivers of procurement, bedside decision-makers (e.g., tests, imaging, and other orders), prescribers, patient educators, mentors to trainees, and advocates. An awareness and understanding of the environmental impact of clinical care is foundational to transforming healthcare delivery and mitigating its carbon footprint, and ultimately ensuring the well-being of patients amid a climate crisis with grave health consequences.

We thank you for taking the time to complete this quality improvement measure on climate change and healthcare sustainability.

- Suggested Reading List: Health Impacts of Climate Change and Pollution
- Suggested Reading List: Healthcare Environmental Impact Including Carbon Footprint
- Educational Seminar Recordings
- MGH Data Dashboard
- Health Care SOS Podcast
- Greening the Research Lab
- Climate Change Measure Video Playlist
- Module slides
    - Climate change impact
    - Healthcare footprint
    - MGH/MGB actions
    - Taking action

© 2024 Armand W et al. *JAMA Network Open.*
